# Introduction

**DOI:** 10.1186/1710-1492-6-S4-A1

**Published:** 2010-12-10

**Authors:** Louis-Philippe Boulet

**Affiliations:** 1 Institut universitaire de cardiologie et de pneumologie de Québec, Laval University Chair on Knowledge Transfer, Education and Prevention in Respiratory and Cardiovascular Health, Québec, Québec, G1V 4G5, Canada

## 

A Clinical Practice Guideline (CPG) can be defined as a “systematically developed statement to assist practitioner and patient decisions about appropriate healthcare for specific clinical circumstances” [1,2]. These guidelines help summarize research findings, provide guidance on management of various conditions, and support quality control measures [3].

They usually include a review of current knowledge, following a systematic search for evidence on a specific topic, and provide graded recommendations to guide the clinician, particularly for primary care settings [4,5]. Unfortunately, guidelines implementation is often still sub-optimal, particularly for chronic diseases [4,6-8]. In the last decade, research in the field of Knowledge Transfer/Translation (KT) has identified possible strategies to improve the uptake of guidelines [9,10].

This report of the proceedings of a Canada-Sweden symposium held in Quebec City, Canada, on 7 May 2010, provides a brief overview of various components of CPG implementation, particularly on allergic respiratory diseases. The main objectives of the symposium were to: 1) Review barriers and facilitators of KT and guidelines implementation for primary care, 2) Discuss current status and recent initiatives in regard to allergy and asthma guidelines implementation in Canada and Sweden, 3) Elaborate on how to improve guidelines implementation at the patient level, for example using shared-decision interventions, and 4) Suggest models of evaluation of KT and implementation programs.

Hopefully, this summary of the presentations will bring useful information to the attention of all those interested in KT and promote new initiatives, particularly for primary care, to improve guideline implementation and evaluation of the results of these interventions.
